# Response of extensively drug resistant *Salmonella* Typhi to treatment with meropenem and azithromycin, in Pakistan

**DOI:** 10.1371/journal.pntd.0008682

**Published:** 2020-10-15

**Authors:** Sonia Qureshi, Abdullah B. Naveed, Mohammad Tahir Yousafzai, Khalil Ahmad, Sarwat Ansari, Heeramani Lohana, Aimen Mukhtar, Farah Naz Qamar

**Affiliations:** 1 Department of Pediatrics and Child Health, Aga Khan University Hospital, Karachi, Pakistan; 2 Medical College, Aga Khan University, Karachi, Pakistan; 3 Department of Pediatric and Child Health, Aga Khan Maternal and Child Care Centre, Hyderabad, Pakistan; Centers for Disease Control and Prevention, UNITED STATES

## Abstract

**Introduction:**

*Salmonella* Typhi is one of the leading health problems in Pakistan. With the emergence of extensively drug resistant (XDR) *Salmonella* Typhi, treatment options are limited. Here we report the clinical manifestations and the response to treatment of patients with XDR Typhoid fever. The patients were treated with either Meropenem or Azithromycin or a combination of both.

**Methods:**

We reviewed the records of culture confirmed XDR typhoid who visited Aga Khan University Hospital (AKUH), Karachi and Aga Khan Secondary Care Hospital, Hyderabad from April 2017 to June 2018. Symptoms developed during disease, unplanned treatment extension and complications developed while on antimicrobials was recorded. Means with standard deviation were calculated for duration of treatment, time to defervescence, and cost of treatment.

**Results:**

Records of 81 culture confirmed XDR typhoid patients admitted at the AKU hospitals were reviewed. Most, (n = 45; 56%) were male. Mean age of the cases was 8.03 years with range (1–40). About three quarter (n = 66) of the patients were treated as inpatient. Fever and vomiting were the most common symptoms at the time of presentation. Oral azithromycin alone (n = 22; 27%), intravenous meropenem alone (n = 20; 25%), or a combination of azithromycin and meropenem (n = 39; 48%) were the options used for treatment. Average (95% confidence interval) time to defervescence was 7.1(5.5–8.6), 6.7(4.7–8.7), and 6.7(5.5–7.9) days for each treatment option respectively whereas there were 1,0 and 3 treatment failures in each treatment option respectively. Average cost of treatment per day for azithromycin was US$5.87 whereas it was US$88.46 for meropenem.

**Conclusion:**

Patients treated with either Azithromycin, Meropenem alone or in combination showed similar time to defervescence. Because of the lower cost of azithromycin, it is preferable in lower socio-economic areas. Background estimates for power calculation can be made for more robust clinical trials using this observational data.

## Author summary

Ever since the emergence of Extensively Drug Resistant (XDR) Typhoid fever in Pakistan, which is resistant to all but two classes of anti-microbials, we need to find an effective treatment plan so that *S*.Typhi does not also make these anti-microbials redundant in the future. In this retrospective case series review, we observed the response of XDR Typhoid fever patients treated with meropenem or azithromycin or combination of both. All 3 groups showed a similar therapeutic response based on number of complications developed and time taken for fever clearance. The higher price of meropenem however could become a deterrent to its use in poor socio-economic areas. This new knowledge may contribute to better treatment plans in handling XDR enteric fever in the future whilst also serving as a rough guide in how to treat patients with XDR Typhoid fever in the present.

## Introduction

Typhoid fever is a systemic infection caused by the *Salmonella* enterica serovar Typhi (*S*.Typhi). According to the World Health Organisation (WHO), estimates of burden of typhoid fever range between 11 and 21 million cases and roughly 128 000 to 161 000 deaths annually[1]. Typhoid fever is a disease of low and middle-income countries but with the emergence of antimicrobial resistance and increase in international travel, the threat is now global[2]. In the last two decades several outbreaks of multi-drug resistant (MDR) *S*.Typhi of H58 genotype have been reported from various parts of the world[3,4]. These organisms are a major threat to typhoid treatment as they are resistant to the first line antimicrobials; ampicillin, chloramphenicol, and trimethoprim-sulfamethoxazole. Similarly, resistance to fluoroquinolones has also increased over the same period. Consequently, third-generation cephalosporins, particularly ceftriaxone, have become the antimicrobials of choice for typhoid treatment in endemic countries[5,6].

In November 2016, a large outbreak of ceftriaxone-resistant typhoid fever started in Hyderabad city of southern Pakistan. The associated organism was a H58 *S*.Typhi exhibiting resistance to five classes of antimicrobials (chloramphenicol, ampicillin, trimethoprim-sulfamethoxazole, fluoroquinolones, and third-generation cephalosporins) and was consequently labeled as extensively drug-resistant (XDR) *S*.Typhi[7,8]. This strain of XDR *S*.Typhi was sensitive to azithromycin and meropenem. The outbreak first started in Hyderabad city with it quickly spreading to the adjacent cities including Karachi. As of August, 2019, more than 10,000 cases of XDR Typhoid have been reported from Hyderabad and Karachi alone[9].

Owing to the novelty of XDR *S*.Typhi, no treatment guidelines are available yet globally. Treatment options are limited and no data on treatment outcomes of XDR Typhoid is available. The data we have is much needed for the development of treatment protocols for XDR Typhoid patients. Moreover, observational data from this study may also be helpful in designing a more robust clinical trial for the treatment of XDR Typhoid in the future.

## Methods

### Study setting and population

A retrospective review of XDR Typhoid cases was conducted in two hospitals; Aga Khan University Hospital (AKUH) in Karachi and Aga Khan Maternal and Child Centre (AKMCC) in Hyderabad. AKUH is a 740-bed private tertiary care teaching hospital located in the metropolitan city of Karachi while AKMCC is a private secondary care hospital catering only to mothers and children, located in the city of Hyderabad. Karachi and Hyderabad are located about 150 kilometers away from each other in the province of Sindh, Pakistan.

**Fig 1:**
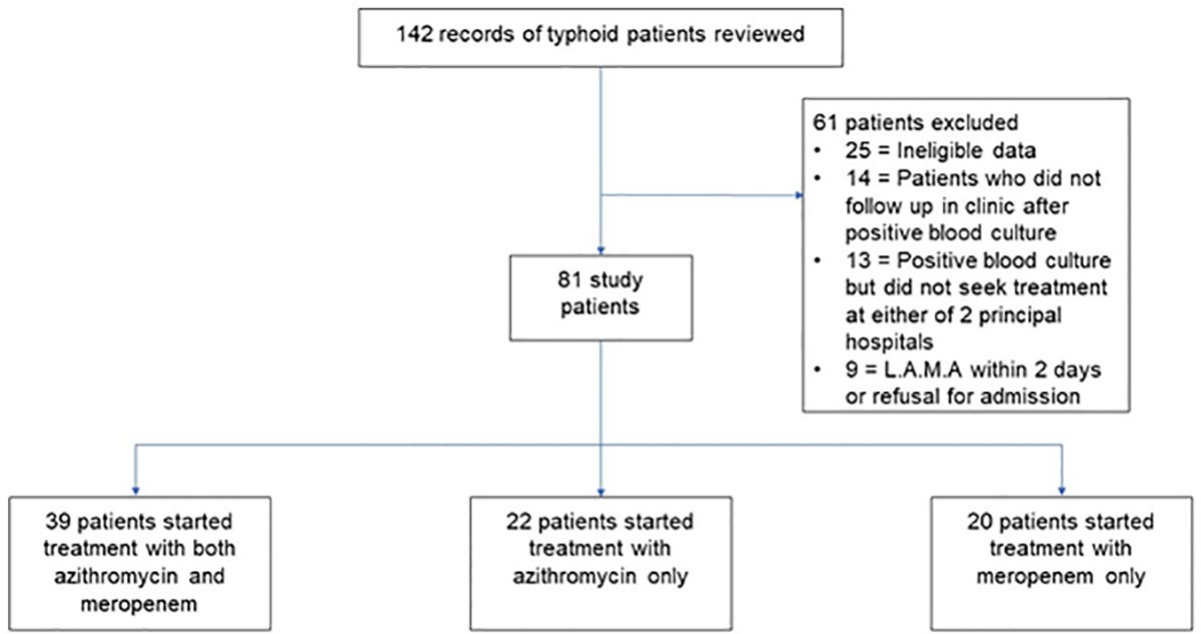
Flow chart of patient inclusion, exclusion and distribution for study population. XDR extensively drug resistant. LAMA, leave against medical advice.

Patients with a diagnosis of blood culture confirmed XDR *S*.Typhi were identified from the registry of both hospitals. Hospital records of typhoid patients treated as either inpatient or outpatient during 1^st^ April 2017 to 30^th^ June 2018 were reviewed for screening to be included in this study. Typhoid patients with blood culture confirmation for *S*.Typhi and resistance to the five classes of antibiotics (ampicillin, chloramphenicol, trimethoprim-sulfamethoxazole, fluoroquinolone, and 3^rd^ generation cephalosporin (ceftriaxone or cefixime) were eligible to be included in this study. Patients with incomplete medical records especially missing information on antimicrobial treatment, duration of treatment, treatment failure, and time to defervescence were excluded from the study. Also, patients who left against medical advice (LAMA) within 2 days of admission, those who had a positive blood culture but did not seek treatment at either hospital or those who did not return for follow-up visit in clinic after the confirmation of blood culture testing were excluded. A total of 142 records with the diagnosis of typhoid were screened and only 81 records which fulfilled the eligibility criteria were included in this study (Fig 1).

### Data collection tool and operational definitions

We used a structured tool in English language to extract information from the medical records. The tool was composed of variables such as demographic characteristics (age, gender), symptoms including fever at the time of presentation to the hospital/clinic, blood culture and antimicrobial sensitivity findings, time in days to defervescence, complications (if any) of disease process, type and duration of antimicrobial therapy, side effects (if any) and treatment failure. Clinical response of antimicrobial therapy was defined as time taken for defervescence (time from first recording of fever, either inpatient or outpatient, till return of body temperature to less than 38^0^ C for more than 48 hours). Treatment failure was defined as either relapse of disease within 30 days of treatment completion, or development of complications while on therapy (septic shock, meningitis, death, hepatitis etc.[10]), or extension of duration of antimicrobial therapy beyond 14 days for meropenem and 10 days for azithromycin. Cost of purchase of anti-microbials was calculated for maximum dosages that could theoretically be given. This meant that meropenem cost was calculated for 2g every 8 hours while azithromycin cost was calculated for 1g every 24 hours. No other direct or indirect costs were measured.

### Ethical considerations

This study was conducted as a part of a larger study which involved investigation of the outbreak of XDR Typhoid and control interventions in Hyderabad and Karachi. The larger study was approved by ethical review committee (ERC) of Aga Khan University Karachi, Pakistan and national bioethics committee (NBC) of Pakistan. For this specific part of the study which involved retrospective review of hospital records, we submitted it for an ERC exemption which was granted.

### Data management and analysis

Two research assistants entered the data independently using the statistical package for social scientists (SPSS) version 25.0. The two datasets were compared to assess for any typographic/data entry errors, validated against the corresponding hard copy of the data tools and corrected. Data was also checked for missing information on the essential variables such as antimicrobial treatment, duration of treatment and time to defervescence. Any variable with extreme values or illogical values were validated against the corresponding physical data tool and the actual medical record files. Frequencies with percentages were calculated for categorical variables such as gender, type of treatment, place (hospital) of treatment, and inpatient versus outpatient treatment. In addition, means with standard deviation were calculated for continuous variables such as age in years, duration of treatment, and time to defervescence etc. Cost of treatment per day for each type of antimicrobial therapy was also calculated and compared across the groups.

## Results

**Fig 2:**
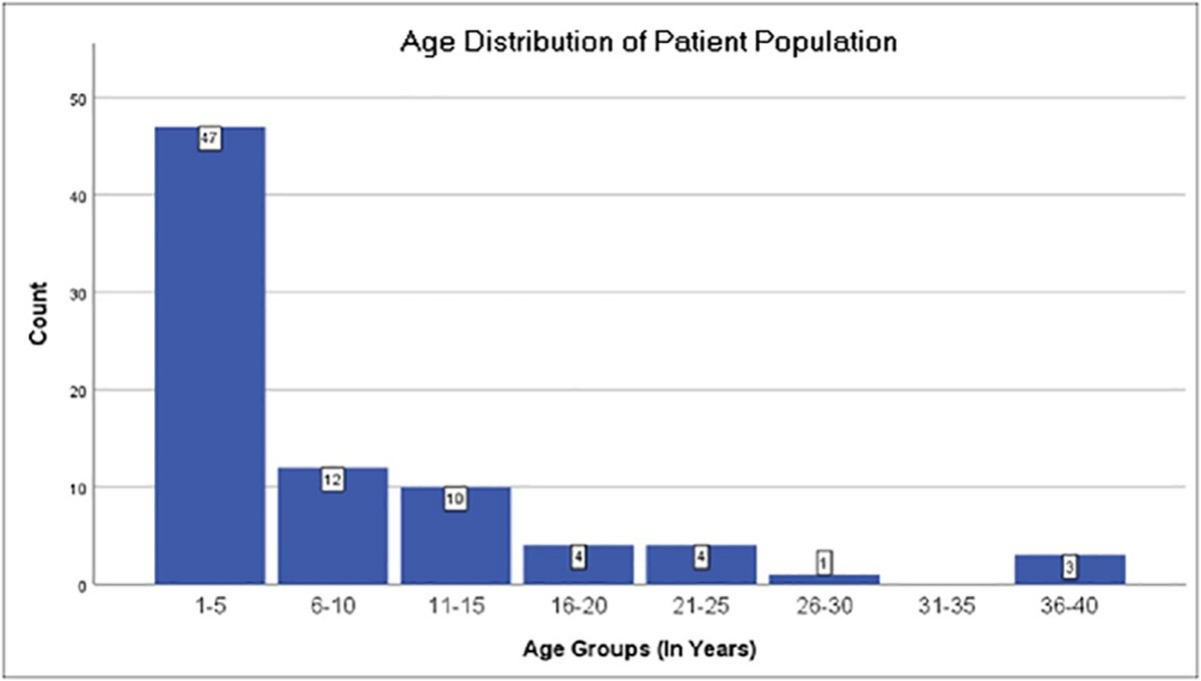
Age distribution of patient population.

Out of 81 records reviewed for this study, 47(58%) were from AKMCC. About three quarter (n = 66; 81%) of XDR Typhoid patients were treated as inpatient. Slightly more than half (n = 45; 56%) of the patients were male and average age of the patients was 8.0 years with range (1–40) (Fig 2). Average temperature at the time of presentation in the hospital was 38.5±0.89 (Range = 40.0–36.0) ^0^C. 64% (n = 52) of patients in this study were started on empirical anti-microbial therapy prior to presentation; either self-prescribed or on the recommendation of a physician (Table 1).

**Table 1:** Demographic characteristics and treatment of culture proven XDR Typhoid patients (N = 81).

Patient characteristics	Frequency (%)
Centers* AKUH AKMCCC	34 (42.0%) 47 (58.0%)
Age in years at diagnosis (Mean) (Min–Max)	8.03 1–40
Gender (Male)	45 (56.0%)
Patients treated as Inpatients	66 (81.0%)
Fever (°C) at time of hospital admission (Mean ± SD)	38.5 ± 0.89
Use of Antibiotics in past 2 weeks	52 (64.2%)
Use of Anti-Pyretics in past 2 weeks	54 (66.6%)
Use of Anti-Diarrheal in past 2 weeks	3 (3.7%)

*AKUH = Aga Khan University Hospital, AKMCCC = Aga Khan Maternal and Child Care Centre Hyderabad.

**Fig 3:**
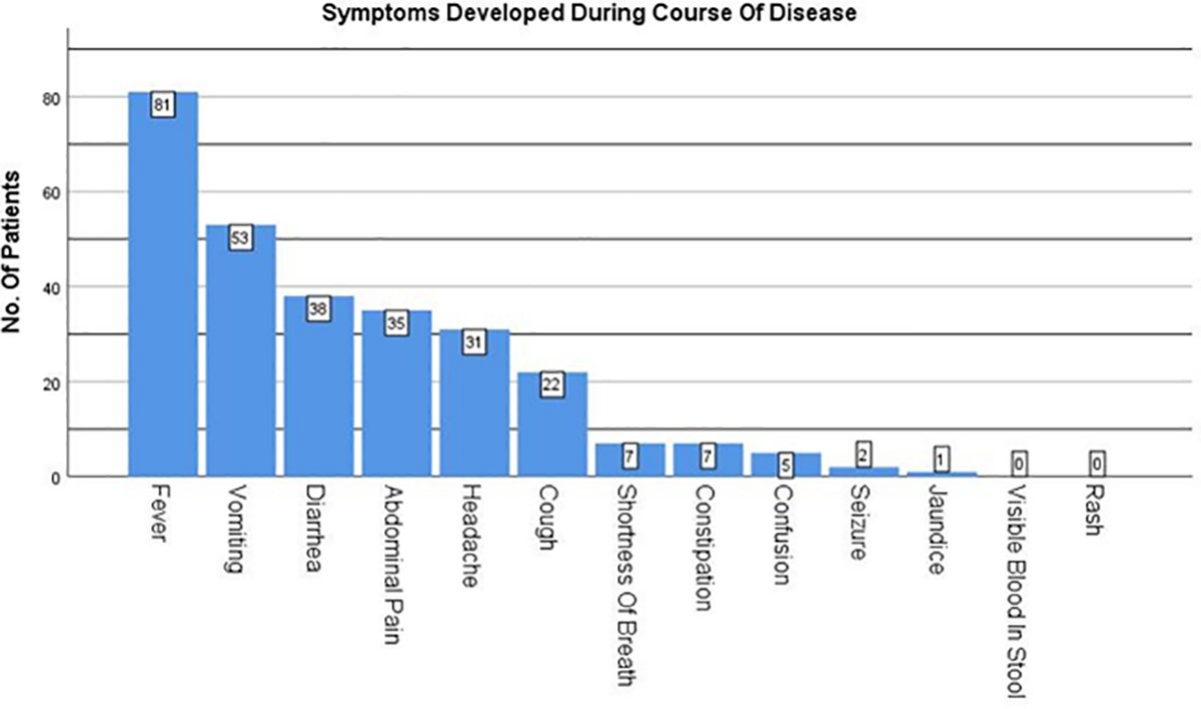
Presenting symptoms of patients at time of hospital encounter arranged in descending order of occurrence.

Fever (n = 81; 100%) and vomiting (n = 53; 65%) were the most common symptoms developed during course of disease, followed by diarrhea (47%), abdominal pain (43%) and headache (38%) (Fig 3**).**

**Fig 4:**
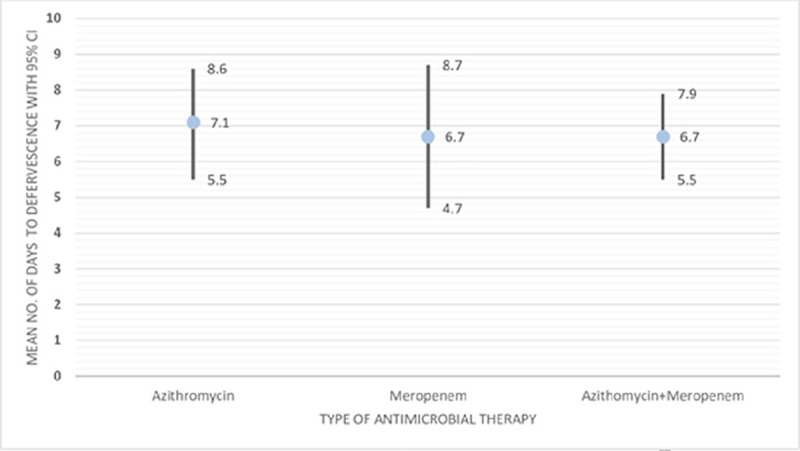
Comparison of time to defervescence across different types of antimicrobial therapy for treating XDR Typhoid patients.

Out of 81 XDR Typhoid patients, 22 (27%) were treated with azithromycin alone, 20 (25%) with meropenem alone and 39 (48%) received a combination of azithromycin and meropenem. Patients who received meropenem only were all treated as inpatient, whereas only 11/22 (50%) patients treated with azithromycin required hospitalization. Meropenem was given Intravenously (IV) at a dose of 20-40mg/kg three times a day while azithromycin was given orally at 20mg/kg/day. There was only one treatment failure among patients treated with azithromycin while on the other hand three treatment failures each were observed among the patients treated with combination of azithromycin and meropenem. No treatment failure was seen in patients treated with meropenem. Average time in days (95% confidence interval) to defervescence was 7.1(5.5–8.6) for azithromycin, 6.7(4.7–8.7) for meropenem, and 6.7(5.5–7.9) for combination of meropenem and azithromycin. Average time to defervescence was lower for the patients treated with meropenem and combination of azithromycin and meropenem as compared to the patients treated with azithromycin (Fig 4). Average daily cost of treatment was highest (US$94.33) for the patients receiving combination of azithromycin and meropenem and lowest (US$5.87) for those treated with azithromycin alone (Table 2).

**Table 2:** Clinical Outcome of patients among the three treatment groups.

Anti-microbial Group	N (%)	Treated as Inpatients n(%)	Treatment Failure n(%)	Duration of Treatment (days) (Mean ± SD)	Cost of Treatment per day ǂ
Meropenem Only	20 (24.7%)	20 (100%)	0 (0%)	8.1 ± 2.5	13800PKR (US$88.46)
Azithromycin Only	22 (27.2%)	11 (50%)	1 (4.5%)	6.6 ± 2.7	915PKR (US$5.87)
Combination of Meropenem and Azithromycin	39 (48.1%)	35 (89.7%)	3 (4.5%)	Azithromycin: 7.5 ± 3.8 Meropenem: 8.5 ± 4.3	14715PKR (US$94.33)

ǂ All prices given in local currency and converted to international dollar according to Bureau of the Fiscal Services Treasury Reporting Rates of Exchange

## Discussion

In this study we assessed the response of azithromycin and meropenem–either alone or in combination, for the treatment of XDR Typhoid fever. All three treatment options were highly effective, with less than 5% treatment failure in each group, however patients treated with meropenem had no treatment failure. Patients treated with meropenem and combination of azithromycin and meropenem had a slightly earlier time to defervescence.

No evidence is available to establish fever clearance time for XDR typhoid. Data from previous studies for MDR typhoid and fluoroquinolone resistant strains, has shown that defervescence is achieved in less than 7 days[11–14]. These results are in line with the expected time to defervescence for non-resistant typhoid fever which is 3.5–8 days from initiation of antimicrobial therapy[15,16]. Our study was consistent with this range and showed all 3 treatment arm groups achieving defervescence within the given range. Meropenem is usually considered as the last option for multi drug resistant gram-negative infections[17] hence there are limited reports that establish a definitive time to fever clearance for typhoid fever when treated with meropenem. Literature search reveals 5 fluoroquinolone resistant *S*.Typhi strains that were treated with meropenem alone [18,19]or as part of combination therapy[20–22]. Out of these, only two of the cases achieved defervescence within the expected range[18,19] when treated with meropenem alone while the remaining three cases required augmentation of meropenem with another antimicrobial to achieve defervescence[20–22]. More robust data is available for azithromycin-based therapies where multiple clinical trials have shown that defervescence is achieved within the expected range [23]. A study by Nair et al suggested that fever clearance time was longer when patients were treated with azithromycin compared to when patients were treated with ceftriaxone[24]. However, in our study, fever clearance time for XDR typhoid fever patients treated with either azithromycin or meropenem or a combination of meropenem and azithromycin was similar.

The treatment failure that occurred in azithromycin only group was because of non-resolving fever even after being given recommended dose and duration of antimicrobial. The fever resolved after extension of antimicrobial therapy by 5 days. No underlying condition (immune suppression) or disease complication or non-compliance to therapy was recorded. The treatment failure percentage with azithromycin in our study is consistent with previous clinical trials that have established the efficacy of azithromycin in treating enteric fever[23]. Frenck et al[25] and Girgis et al[26] explained that the intracellular accumulation of azithromycin within cells, its secretion into the biliary tree and the long half-life of the drug make it extremely effective against intracellular infections like enteric fever. Literature, however, is inconclusive when it comes to the efficacy of meropenem for treating enteric fever. Four case reports point to its clinical inefficacy based on either relapse of disease[19] or augmentation of meropenem with another antimicrobial due to unsatisfactory clinical results with meropenem alone[20–22]. Godbole et al[20] discussed the possible treatment failures due to the limited intracellular penetration of meropenem whereas Blumentrath et al[19] explained the treatment failures via the phenomena of tolerance and persistence exhibited by *S*.Typhi. In contrast, Munir et al[18] showed favorable results when treating XDR Typhoid fever with meropenem. Our study patients showed adequate results when treated with meropenem. Meropenem is a type of *β*-lactam antibiotic with inherent stability against ESBLs and possesses a broad-spectrum activity, these factors could explain the clearance percentage for meropenem[27]. In the group treated with combination of meropenem and azithromycin, there were 3 treatment failures. All 3 patients developed systemic complications and unfortunately, one of the patients passed away. This could be attributed to delay in treatment of both patients because of delayed presentation to the hospital resulting in an unfavorable clinical picture on arrival. The preference (n = 39; 48%) for using both antibiotics together can likely be explained by the fact that one is a bacteriostatic (meropenem) and the other is a bactericidal (azithromycin). Past studies have shown that the combination of ceftriaxone (a bactericidal) with azithromycin (a bacteriostatic) reduced fever clearance times[28], albeit no study could be found assessing the synergistic action of meropenem with azithromycin for treating Enteric fever. Case reports by Kleine et al[21], Godbole et al[20] and Blumentrath et al[19] also suggested that meropenem be combined with another antimicrobial agent for treating fluoroquinolone resistant and XDR typhoid fever. Since there are no evidence based clinical guidelines for the treatment of XDR Typhoid, azithromycin is a good choice since it accumulates intracellularly and has a long half-life.

The longer duration of treatment with azithromycin (6.6 ± 2.7 days) in our study compared to established protocols[23] was likely due to treating physicians fearing a relapse of the disease and hence giving extended doses of azithromycin. The 1 treatment failure which required extension of antimicrobial therapy in this group also slightly skewed the mean. For meropenem however, there are only limited case reports that guide us about the dosing and duration of meropenem for XDR typhoid fever or fluoroquinolone resistant typhoid fever. Blumentrath et al[19] administered it for 10 days where as Kleine et al[21] had to administer it for 22 days which shows the variance in time meropenem takes to achieve fever clearance. The duration of treatment with meropenem in our study was similar to duration of treatment of Typhoid fever with ceftriaxone in past clinical trials[29]. We believe this was due to the similar mechanism of action of the two antimicrobials (both are bactericidal) and short half-life’s (60mins for meropenem vs 330mins for ceftriaxone)[30][31].

Another facet to consider from this study is the cost of therapy of meropenem compared with azithromycin. Enteric fever is endemic in countries with a lower socio-economic status due to difficulty in maintaining hygienic conditions. Patients on meropenem therapy must incur an average daily cost of US$88.46 compared to US$5.87 for azithromycin. For patients with a lower socio-economic status, the extra cost can prove to be a deterrent in obtaining the optimum therapy.

Since this is a retrospective study, the information on patient follow ups is limited and the detailed cost of hospitalization was not recorded. The criteria for assessing which patient necessitated a specific therapy was also subjective and based on the discretion of the treating physician Also, we believe the study might have had selection bias because of patients who were lost to follow up and those who were excluded based on lack of proper data. Furthermore, as there is an uneven receipt of antibiotics received before presentation to hospital, the average time to defervescence could have been affected across groups, though none of them had either received azithromycin or meropenem. The strength of this study was the inclusion of cases based only on positive blood cultures and not clinical judgement.

In conclusion either azithromycin or meropenem alone or in combination were used for the treatment of XDR Typhoid in an outbreak setting in Pakistan. The time to defervescence and the percentage of treatment failures in all groups were similar. Azithromycin is a convenient choice based on cost of therapy and availability in oral form. Clinical trials are needed to establish days of treatment required and the best treatment options for XDR Typhoid fever.

## Supporting information

S1 Supporting Information STROBE checklist. (DOCX)
